# Supplementary material for: Improving the understanding of cytoneme-mediated morphogen gradients by in silico modeling
Source: PLoS Comput Biol. 2021 Aug 3;17(8):e1009245. doi: 10.1371/journal.pcbi.1009245 (PMC8362982; doi:10.1371/journal.pcbi.1009245)
Supplement: S1 Text — (DOCX) [file pcbi.1009245.s001.docx]

**The dynamics of cytoneme lengths**

$\lambda_{r}(t)$and $\lambda_{p}(t)$ describe the dynamics of elongation and retraction of cytonemes emanating from either receiving $x_{r}$ or producing $x_{p}$cell positions. It has been experimentally demonstrated that cytonemes have different dynamics (1): they not only elongate and retract (Triangular behavior, Fig 1E), but also have intermediate stationary phases, during which cytonemes maintain their maximum elongation (Trapezoidal behavior, Fig 1F). Those behaviors have been implemented in this work as follows:

$$Triangular dynamics:$$

$\lambda^{triangle}(t)=\left\{ \begin{aligned} v_{e}^{triang}\cdot t if 0\leq t\leq t_{e}^{triang} \\ v_{rt}^{triang}\left( t-t_{e}^{triang} \right) +\lambda_{max} if t_{e}^{triang}<t\leq t_{\tau}^{triang} \end{aligned} \right.$ (S1-eq 1.1)

$$Trapezoidal dynamics:$$

$$\lambda^{trapezoid}\left( t \right)=\left\{ \begin{aligned} v_{e}^{trap}\cdot t if 0\leq t< t_{e}^{trap} \\ \lambda_{max} if t_{e}^{trap}\leq t< t_{s}+t_{e}^{trap} \\ v_{rt}^{trap}\left( t-(t_{s}+t_{e}^{trap}) \right)+ \lambda_{max} if t_{s}+t_{e}^{trap}\leq t< t_{\tau}^{trap} \end{aligned} \right.$$

(S1-eq 1.2)

Where $v_{e}$ and $v_{rt}$ are the velocities of elongation and retraction for Triangular of Trapezoidal dynamics (described by superscripts *triang* and *trap* respectively). Both variables include the sign, being $sgn\left( v_{e} \right)=+1$ and $sgn\left( v_{rt} \right)=-1$.

$t_{e}$ is the time spent by a cytoneme in the elongation phase and$t_{\tau}$ is the total time from elongation to retraction; $t_{\tau}$ is defined as $t_{\tau}$ =$t_{e}$ +$t_{rt}$ for triangles and $t_{\tau}$ =$t_{e}$ +${t_{s}+t}_{rt}$ for trapezoids, ${t_{s}, t}_{rt}$ being the time spent in the stationary and retraction phases respectively. $\lambda_{max}$is the maximum length of a cytoneme (elongating with $v_{e}$ during $t_{e}$).

Expressions S1-eq 1 are defined for a time variable $t\in(t_{0}, t_{\tau})$ with initial time $t_{0}=0$. In the code, cytonemes independently elongate and retract multiple times throughout the entire simulation with a homogeneous frequency determined by the parameter “temporal density” in the GUI; to this end, $t_{aux}$ (an auxiliary temporal variable) was introduced in the code for the S1-eq 1 equations.

The design of the temporal dependence of the contact functions (Eq-3) through the functions $\lambda(t)$ allows the introduction of different cytoneme dynamics, including the static case: $\left\{ \begin{aligned} \lambda_{r}\left( t \right)=\lambda_{r} \\ \lambda_{p}\left( t \right)=\lambda_{p} \end{aligned} \right.$

**Supplementary references**

1. González-Méndez L, Seijo-Barandiarán I, Guerrero I. Cytoneme-mediated cell-cell contacts for hedgehog reception. Elife. 2017 Aug 21;6.
