# Supplementary material for: Improving the understanding of cytoneme-mediated morphogen gradients by in silico modeling
Source: PLoS Comput Biol. 2021 Aug 3;17(8):e1009245. doi: 10.1371/journal.pcbi.1009245 (PMC8362982; doi:10.1371/journal.pcbi.1009245)
Supplement: S2 Text — (DOCX) [file pcbi.1009245.s002.docx]

**The probability of contacts** $\boldsymbol{\psi}\left( \boldsymbol{\mu,x} \right)$

The cellular mechanisms behind the establishment of cytoneme contacts are not well understood. We took advantage of the capability of Cytomorph to simulate situations difficult to study experimentally to predict the effect on the gradient of different types of contact functions $\psi\left( \mu,x \right)$. As an example, in what follows we describe the information obtained about this function looking at its dependence on different variables.

1. If $\psi\left( \mu,x \right)$ does not depend on cell position, then $\psi\left( \mu,x \right)=\psi\left( \mu\right).$ This implies that cytonemes always stablish contacts with a probability $\mu$, if they satisfy the distance condition needed to find another cell membrane (Eq-3). In this case, the probability of contact can be defined as the efficiency to establish connection for morphogen release.

$\psi\left( \mu\right)= \left\{ \begin{aligned} 1 with a probabilty of \mu\\ 0 with a probabilty of 1-\mu\end{aligned} \right.$ (S2-eq 1.1)

Cytonemes overlapping for long stretches have experimentally been observed in *Drosophila* wing disc (1). This can be modeled as a function independent of cell position assuming that new contacts along the overlapping stretch are more likely to occur after an initial contact between two cytonemes. For type 3 experimental case we developed a different module in the software in which the probability of function for $C_{3}\left( x_{r} \right)$has been defined as follows:

$$\Psi\left( \mu\right)=\psi\left( \mu\right)+\delta_{\psi\left( \mu\right) , 1}\sum_{i=1}^{\lambda_{overlap}} \psi\left( \mu_{i} \right)$$

$with \mu_{i}=\left\{ \begin{aligned} \mu+\left( r\cdot\mu\right)\cdot i if \mu+\left( r\cdot\mu\right)\cdot i \leq1 \\ 1 if \mu+\left( r\cdot\mu\right)\cdot i > 1 \end{aligned} \right.$ and $\mu\in[0,1]$

(S2-eq 1.2)

The contact probability for the overlapping $\mu_{i}$ increases with the number of previous contacts (i) in a rate $r$ that has to be determined experimentally (simulations in this work used $r=0.2$).

Note that the single contact function is the same as in S2-eq 1.1, with the exception that the delta of Kronecker makes the sum different from zero only when a previous contact has been established. The sum depends on the integer overlapping length $\lambda_{overlap}$, which is a dynamic value determined by $\lambda_{overlap}\left( t \right)=\lambda_{r}\left( t \right)+\lambda_{p}\left( t \right)-x_{r}-x_{p}$.

1. If $\psi\left( \mu,x \right)$ depends on the cell position within the tissue, this probability variable is related with the weight functions used in other models. These weight functions might indicate the existence of cellular mechanisms that control the establishment of a contact with a specific cell to regulate the amount of transferred morphogen. This spatial dependence could also be related to the accessibility of the receiving cell, since cytonemes have to navigate through a tissue evading biochemical, mechanical and physical constraints.

In both possibilities the function can be written as:

$\psi\left( \mu,x \right)=\psi\left( \mu(x) \right)=\left\{ \begin{aligned} 1 with a probabilty of \mu(x) \\ 0 with a probabilty of 1-\mu(x) \end{aligned} \right.$ (S2-eq 1.3)

Where the function $\mu\left( x \right)$could depend on the cellular mechanisms or on the tortuosity of the path. Since these possibilities are very specific of either the tissue or the path, in this work we implemented a simple case in which the probability decays linearly as $\mu=m\cdot x_{r}+n$.

The parameters m and n were calculated using the following conditions:

- The probability of contact $\mu$ is zero in regions more distant that the sum of the maximum elongation lengths of cytoneme protruding from the receiving and producing cells.

$$\mu\left( \lambda>\lambda_{r}+\lambda_{p} \right)=0$$

- The probability function of contacts is normalized to 1.

$$\int_{0}^{\lambda_{r}+\lambda_{p}} \mu(x_{r})dx_{r}=1$$

Therefore, the final form used for the variable probability along the receiving cells was:

$\mu(x_{r})=\frac{2}{{(\lambda}_{r}+\lambda_{p})}\left[ \frac{-x_{r}}{{(\lambda}_{r}+\lambda_{p})}+1 \right]$ (S2-eq 2)

**Supplementary references**

1. González-Méndez L, Seijo-Barandiarán I, Guerrero I. Cytoneme-mediated cell-cell contacts for hedgehog reception. Elife. 2017 Aug 21;6.
