# Supplementary material for: Improving the understanding of cytoneme-mediated morphogen gradients by in silico modeling
Source: PLoS Comput Biol. 2021 Aug 3;17(8):e1009245. doi: 10.1371/journal.pcbi.1009245 (PMC8362982; doi:10.1371/journal.pcbi.1009245)
Supplement: S3 Text — (DOCX) [file pcbi.1009245.s003.docx]

**Cytomorph software architecture**

Cytomorph was designed in different scripts that can be divided into the following modules (Fig A):

1. A module containing a group of scripts for the graphical user interfaces (GUIs) was designed to run simulations.
2. A module to numerically simulate the cytoneme dynamics and to compute contacts and their spatial distribution over time.
3. A module to plot graphs to visualize the simulated spatial and temporal contact distribution and to understand the effects of different cytoneme features on gradient properties.

1*. Graphical user interface (GUI)*

The GUI is composed of 4 different scripts to create the graphical windows. ***Cytomorph*** is the main script and contains the code for the initial window (Fig B) in which experimental data can be loaded (via Table A) and cytoneme features can be selected. If we want to compute different simulations and compare them, ***Cytomorph*** calls to the script ***cases2compare*** that creates a secondary window (Fig 2B-1). In this window different combinations of parameters (cases) can be loaded to compare with the initial case, taken as reference. For convenience, if the cases to compare are just different values (scan) of the same variable, we also created a third script called ***Scan*** that generates a window (Fig 2B-2) in which a scan of a variable value can be determined. Finally, there is another script called ***NewGraph*** that generates a window (Fig 2B-3) in which the graphical properties can be selected if the user wants different options of default properties.

After collecting all the parameters, the main script ***Cytomorph*** calls the computing module to run the corresponding experimental data and the selected features to be studied.

2. *Numerical computation*

This module numerically simulates the cytoneme dynamics and computes the contacts and their distribution in the receiving cells for the parameters selected and for the experimental data loaded.

This module is made up of 6 different scripts, controlled by a main script (called ***CytomorphFunction***), which receives the parameters from the graphical module and calls the script functions according to the properties to be simulated.

If the case “dynamic cytonemes” is selected, then ***CytomorphFunction*** calls for the dynamic module. This module computes the sum of the possible contact distributions in the receiving cells, determined according to Eq-3 and S1-eq 1.

Since the mechanisms that influence cytoneme dynamics are not well understood, some hypotheses can be verified, such as the effect of the type of contact function $\psi\left( \mu,x \right)$. To this end we created a variable called CL; if selected (CL=1), the ***CytomorphFunction*** calls the script ***DynamicCL*** to compute the contact according to S2-eq 1.2. Otherwise (CL=0), the main script calls the script ***Dynamic*** that computes the contact distribution according to S2-eq 1.1, if the probability $\mu$ is a constant (case $\psi\left( \mu,x \right)=\psi\left( \mu\right)$), or to S2-eq 1.3, if the probability is a function of the position (case $\psi\left( \mu,x \right)=\psi\left( \mu(x) \right)$).

Computationally $\psi\left( \mu\right)$ is coded by the function randsrc in Matlab as follows:

$$\psi\left( \mu\right)=randsrc( 1, 1,\left[ 0 1;1-\mu\mu\right])$$

We observed that the computational runtime cost of this function of our code is high compared to other code sections. For convenience, if we are working with a contact probability of 100% (μ = 1), to reduce the simulation time we created two more scripts called ***DynamicProb100*** and ***DynamicCLProb100*** in which we replaced function randsrc for $\psi\left( \mu\right)=1$.

Finally, to also consider static cytonemes, we created a static module that computes the sum of all possible contact distributions in the receiving cells, determined according to Eq-3 for static cytonemes ($\lambda_{r,p}(t)$ =$\lambda_{r,p}=$const). As in the dynamic case this module is divided in two different scripts: ***Static*** (CL=0) and ***StaticCL*** (CL=1) that compute the contact distribution according to the different S2-eq 1 equations.

*3. Plotting module*

While the main computation was done in the previous modules, all the information of the contact distribution was stored in an array to be subsequently used in the plotting module to depict different features that help the understanding of the software results.

This module is a main script called ***SimulationPlots*** in which the simulation results are graphically plotted in figures to facilitate the interpretation of the numerical simulations of our code. The selected magnitudes that Cytomorph plots as outputs are estimated as follows:

- *Contact distribution*

The software computes the number of contacts per cell along the simulated time. This is repeated over a wide range of simulations per case and the resulting contact distribution array is the base for computing the rest of the parameters. Since the software collects a random subgroup from the published experimental data values (1) in each simulation, we can simulate the number of contacts of a specific receiving cell position ($x_{r})$and see the predicted value per simulation (Fig 2C-1). To visualize the contact distribution along simulations violin plots are shown in Fig 2C-2.

- *Signal variability*

The predicted values for simulation variability can be determined from the different simulations. To better study this parameter and statistically compare different cases, we computed the distribution of coefficients of variation per case. To do this, we divided the N simulations runs into 100 subgroups of N/100 samples each. Then, the coefficient of variation distribution per case was performed over those 100 subgroups. The resulting data are presented as violin plots in Fig 2C-3. (Note: if the number of simulation runs is lower than 1000 (N<1000), then the division is done into 10 subgroups of N/10 samples each).

- *Temporal evolution*

We can also observe the number of contacts in each receiving cell per time lapse (Fig 2C-4) and the total evolution of the contact distribution and gradient shape over the simulated signaling time (Fig 2C-5).

- *Gradient distribution*

The software computes the equations in each iteration and subsequently, for a validation of the model, the software plots the simulation versus the experimental data of the morphogen gradient according to equations in S4 text. In this way, we can visualize the accuracy of the *in silico* predictions.

As mentioned before, the software gives a numerical estimation for the number of contacts per receiving cell along time. The plotted gradient (Fig 2C-6) is a normalized gradient calculated from equations in S4 text; specifically, it is the normalized exponential fit of the numerical simulations, together with the standard deviation of the simulations performed per case (in error bars).

This plotting module also contains the next two auxiliary scripts:

***Violin:*** Since Violin plots are not implemented in the Matlab base code, we externalized these plots in the software using the script developed by Holger Hoffmann, which is available in MatlabWorks:

*Hoffmann H, 2015: violin.m - Simple violin plot using matlab default kernel density estimation. INRES (University of Bonn), Katzenburgweg 5, 53115 Germany.*

<https://es.mathworks.com/matlabcentral/fileexchange/45134-violin-plot>

***swtest***: Since Shapiro-Wilk test is not implemented in Matlab code, for the statistical study of normality we used the script developed by Ahmed Ben Saïda, which is available in MatlabWorks:

*Copyright (c) 17 March 2009 by Ahmed Ben Saïda,*

*Department of Finance, IHEC Sousse – Tunisia*

<https://es.mathworks.com/matlabcentral/fileexchange/13964-shapiro-wilk-and-shapiro-francia-normality-tests>

**Supplementary references**

1. González-Méndez L, Seijo-Barandiarán I, Guerrero I. Cytoneme-mediated cell-cell contacts for hedgehog reception. Elife. 2017 Aug 21;6.


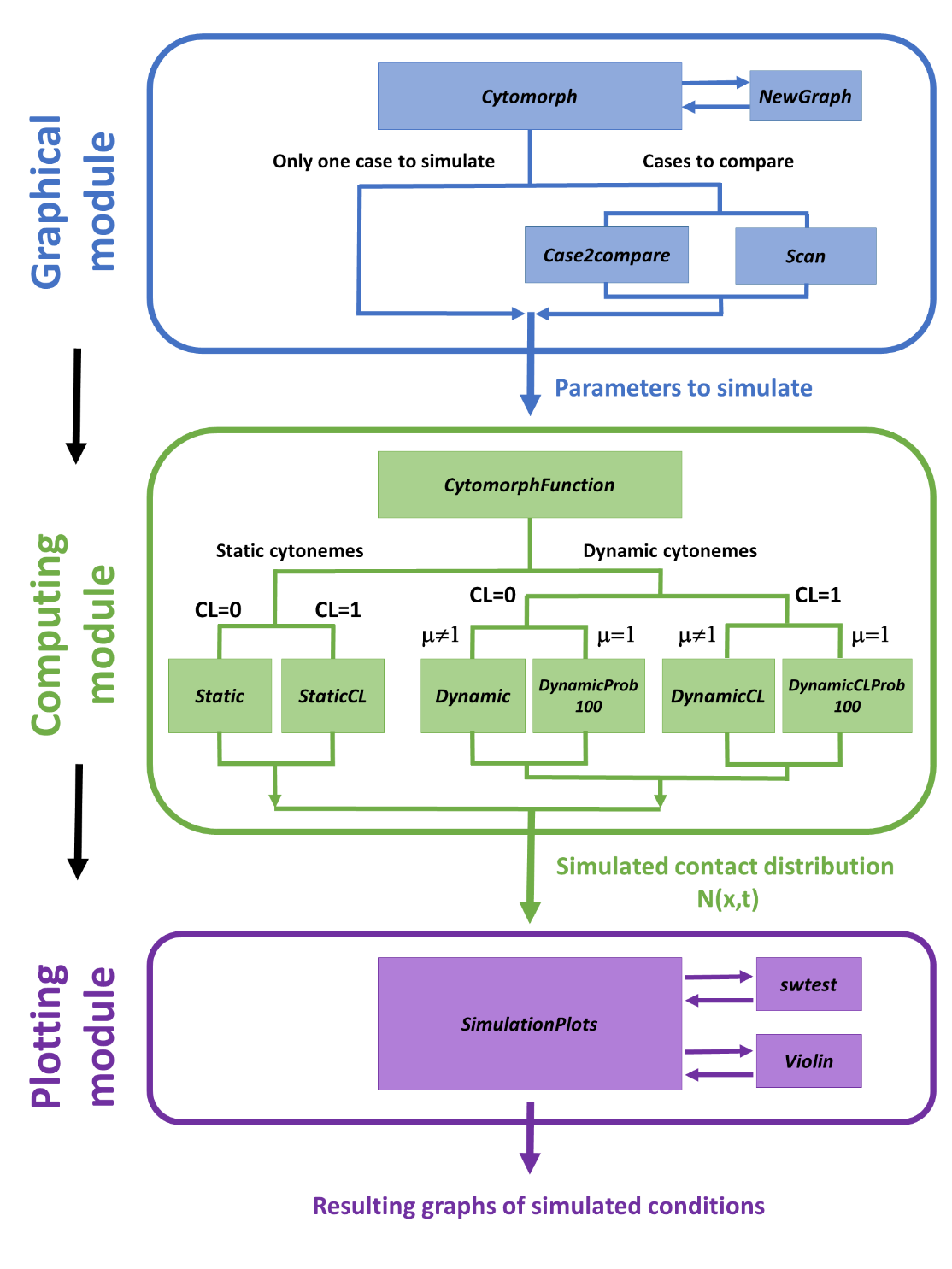


**Fig A. Schematic view of computational steps of Cytomorph machinery.** Cytomorph is composed by 14 different scripts that can be divided in three groups according to their function: In blue, scripts that code the graphical user interphase. In green, scripts that compute the dynamic of cytonemes and their contacts depending on different variables (static or dynamic cytonemes, if cytonemes can contact along their length (CL=1) or not (CL=0) and probability of contacts μ). In purple, the scripts that plot the numerical simulations.


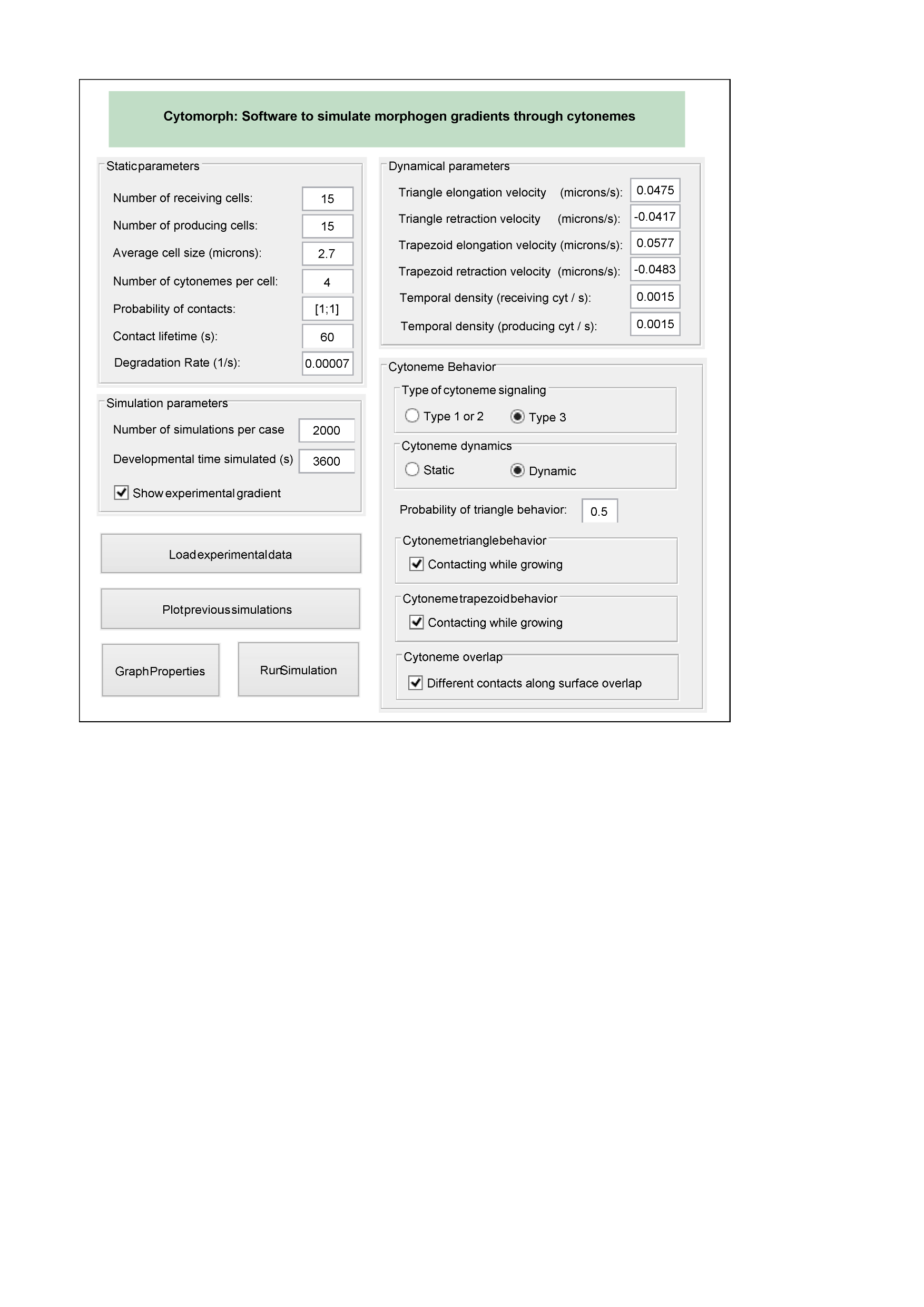


**Fig B. Main window of Cytomorph software.** Detailed view of the main GUI window of Cytomorph, in which different *in silico* conditions can be selected to study and simulate (with their respective units in brackets).

**Table A. Model inputs through** **spreadsheet.** Distribution of parameters (with their respectively units) that should be updated by an Excel file to Cytomorph.
