# Supplementary material for: Improving the understanding of cytoneme-mediated morphogen gradients by in silico modeling
Source: PLoS Comput Biol. 2021 Aug 3;17(8):e1009245. doi: 10.1371/journal.pcbi.1009245 (PMC8362982; doi:10.1371/journal.pcbi.1009245)
Supplement: S4 Text — (DOCX) [file pcbi.1009245.s004.docx]

**Comparing experimental and *in silico* morphogen gradients.**

The concentration of morphogen released in each cell can be determined by the general equation:

$[protein]=F(contact)-\delta\cdot[protein]$ (S4-eq 1)

where the function $F$ determines the quantity of morphogen transmitted in each contact; it depends on biological variables that control the morphogen transmission in the contact place. In this work, we assumed the all contacts are similar and therefore, each contact contributes with the same amount of morphogen. In this case, the function is the number of contacts per cell multiplied by a constant:

$$F\left( contact \right)=\alpha\cdot\#contact$$

As we want to compare the simulations with experimental data, we have first analyzed under which conditions it is possible to compare the following independent normalizations: the data of experimental fluorescent intensity (normalized to the maximum intensity $I_{max}$) versus the contact function in the theoretical model (normalized to the maximum contact function ${\#contact}_{max}$).

The equation S4-eq 1 in a certain period of time $\left[ t_{i},t_{i+1} \right]$ can be written as:

$[protein]=\alpha\cdot\#contact-\delta\cdot[protein]\cdot\left( t_{i+1}-t_{i} \right)$ (S4-eq 2)

If we normalize the equation S4-eq 2 to the maximum concentration of morphogen we obtain:

$$\frac{[protein]}{{[protein]}_{max}}=\frac{\alpha\cdot\#contact}{{[protein]}_{max}}-\frac{\delta\cdot[protein]\cdot\left( t_{i+1}-t_{i} \right)}{{[protein]}_{max}}$$

$$\frac{[protein]}{{[protein]}_{max}}+\frac{\delta\cdot[protein]\cdot\left( t_{i+1}-t_{i} \right)}{{[protein]}_{max}}=\frac{\alpha\cdot\#contact}{{[protein]}_{max}}$$

$(1+\delta\cdot\left( t_{i+1}-t_{i} \right))\cdot\frac{[protein]}{{[protein]}_{max}}=\frac{\alpha\cdot\#contact}{{[protein]}_{max}}$ (S4-eq 3)

Since it is a linear proportion ($\alpha$ = constant), the maximum intensity corresponds to the maximum number of contacts:

$${[protein]}_{max}=\alpha\cdot{\#conctact}_{max}-\delta\cdot{[protein]}_{max}\cdot\left( t_{i+1}-t_{i} \right)$$

This can be approximated to ${[protein]}_{max}\approx\alpha\cdot{\#conctact}_{max}$ in the region of the maximum value, since we can consider $\alpha\cdot{\#conctact}_{max}\gg\delta\cdot{[protein]}_{max}\cdot\left( t_{i+1}-t_{i} \right)$.

Therefore, S4-eq 3 can be written as:

$\left( 1+\delta\cdot\left( t_{i+1}-t_{i} \right) \right)\frac{\left[ protein \right]}{\left[ protein \right]_{max}}\approx\frac{\alpha\cdot\#contact}{\alpha\cdot{\#conctact}_{max}}=\frac{\#contact}{{\#conctact}_{max}}$ (S4-eq 4)

Since the fluorescence intensity of a GFP protein tagged to the morphogen (*Hh:GFP BAC*) was used to obtain the experimental data, the amount of protein is related to the intensity as $I=\beta\cdot\left[ protein \right]$; then, if we normalize to the maximum intensity $I_{max}$ :

$$\frac{I}{I_{max}}=\frac{\beta\cdot[protein]}{\beta\cdot{[protein]}_{max}}=\frac{[protein]}{{[protein]}_{max}}$$

It is important to mention that the previous linear relation is only valid if the acquisition of image samples by confocal laser microscopy has been taken without reaching saturation and with a linear gamma function of acquisition.

Considering the previous confocal conditions, the normalization to the maximum contact in equation S4-eq 4 can be compared with the experimental data (normalized to $I_{max}$) using the relation:

$(1+\delta\cdot\left( t_{i+1}-t_{i} \right))\frac{I}{I_{max}}\approx\frac{\#contact}{{\#conctact}_{max}}$ (S4-eq 5)

Therefore, the conditions to compare *in silico* simulations with experimental data are:

- Mathematically: The degradation rate of the morphogen should be taken into account using S4-eq 5.
- Experimentally: Confocal images must have been taken according to the linear gamma function and within the limits of the acquisition range.
