# Supplementary material for: Improving the understanding of cytoneme-mediated morphogen gradients by in silico modeling
Source: PLoS Comput Biol. 2021 Aug 3;17(8):e1009245. doi: 10.1371/journal.pcbi.1009245 (PMC8362982; doi:10.1371/journal.pcbi.1009245)
Supplement: S5 Text — (DOCX) [file pcbi.1009245.s005.docx]

**Variability and fluctuations in Cytomorph.**

The inputs for the model via spreadsheet are the distributions of cytoneme lengths and elongation, stationary and retraction times for Triangle and Trapezoid cytoneme dynamics. Cytomorph randomly selects a subset of these data distributions for each simulation; this, together with the probability of contacts, generate small differences in the contact distribution for the simulated conditions, which is the source of variability and fluctuations in the model.

The variability of the model has been studied computing different parameters for each cell position (absolute number of contacts, relative number of contacts and coefficient of variation), developing different graphs to visualize the rough data (Figs A-B) and plotting the summarized data in violin plots (Figs C-D).

We were also concerned about the similarity between the predicted variability and the one experimentally observed. To compare with the measured experimental variability, in the final graphical representation of the gradient for each simulated condition we included the simulated variability (numerical standard deviation plotted in error bars)

In this work, we have also paid special attention to how this variability can be altered by different cytoneme features. Since we had observed that the relative variation in the numbers of contacts (standard deviation at position *x* divided by the mean number of contacts for that position) follows a general tendency to grow with receiving cell position (Fig E), we selected the first cell row as the reference position to study the change in variability between different conditions. For that purpose, we have used graphs (Fig A-D) and statistical tests (p-value matrices) as described in a previous section.

The largest fluctuations were found in the tail of the morphogen gradient (last rows of receiving cells). Since they correspond to the region in which the amount of morphogen is low, the fluctuations do not change the activation of the low-threshold targets like Cubitus interruptus (Ci). We then concluded that those fluctuations were biologically negligible for our study.


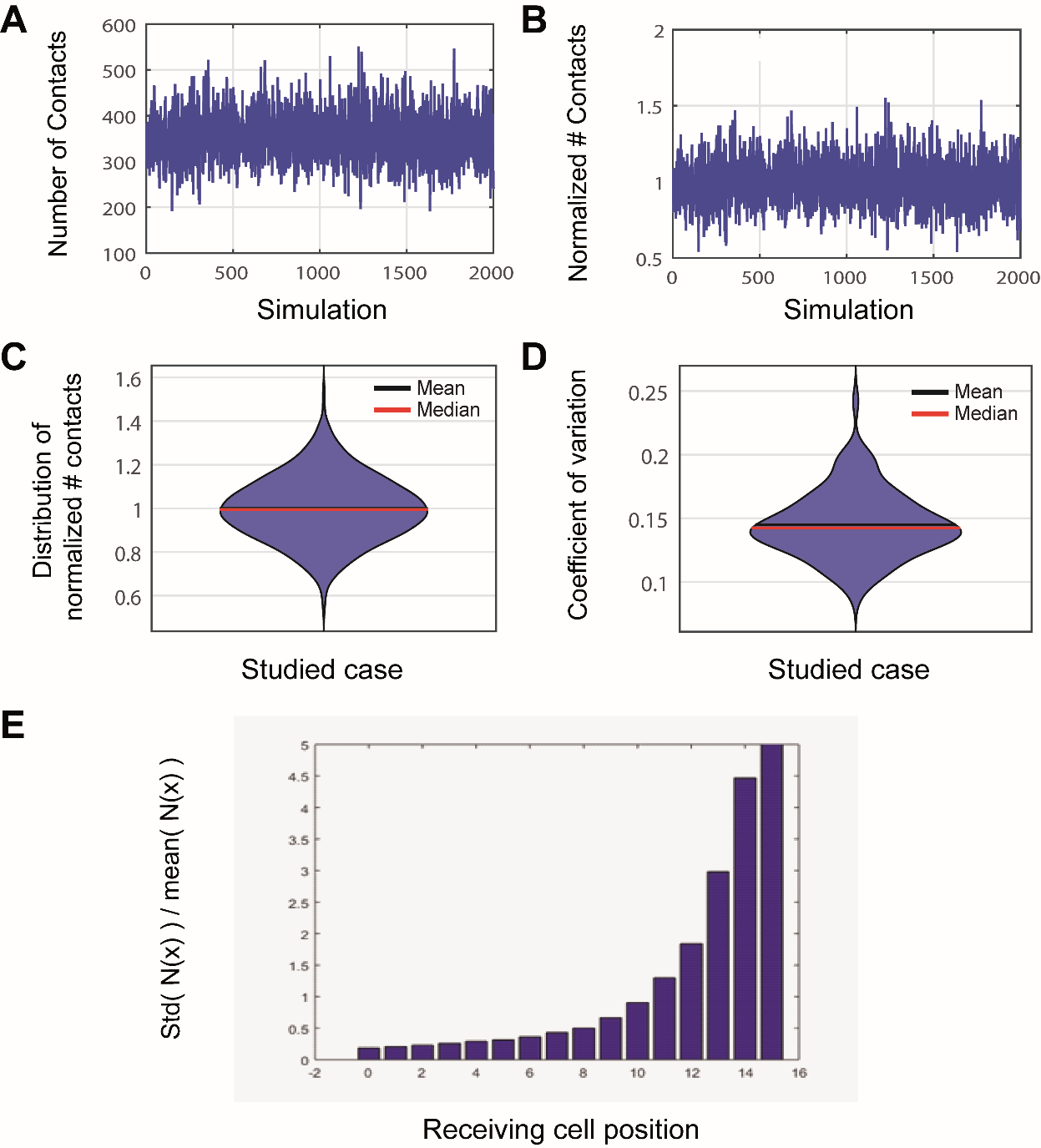


**Fig. Study of the variability and fluctuations in the model.** The figure shows different representations of the variability simulated by Cytomorph: A) Total number of contacts per simulation in the first cell row, $x_{0}$. B) Number of contacts per simulation in the first cell row ($x_{0})$ normalized to the mean value over all simulations. C) Violin plot of data distribution in the image B. D) Violin plot of the coefficients of variation of the previous data. E) Simulated relative variability per cell position: ($std(N\left( x \right))/mean(N(x)$).
