# Supplementary figures and images for: Improving the understanding of cytoneme-mediated morphogen gradients by in silico modeling
Source: PLoS Comput Biol. 2021 Aug 3;17(8):e1009245. doi: 10.1371/journal.pcbi.1009245 (PMC8362982; doi:10.1371/journal.pcbi.1009245)

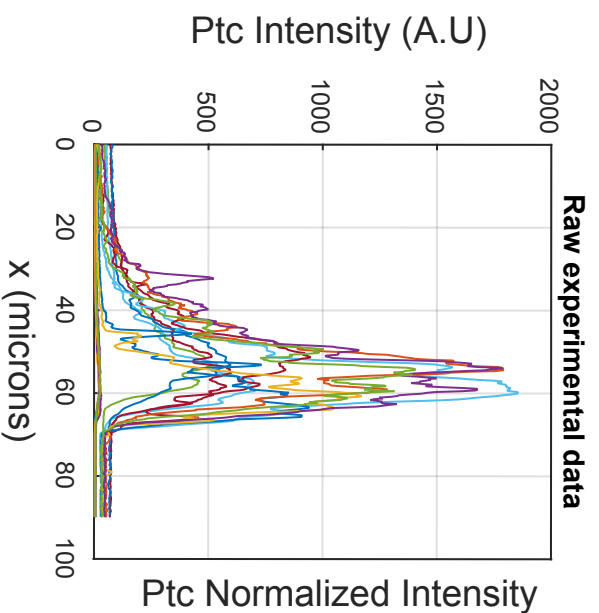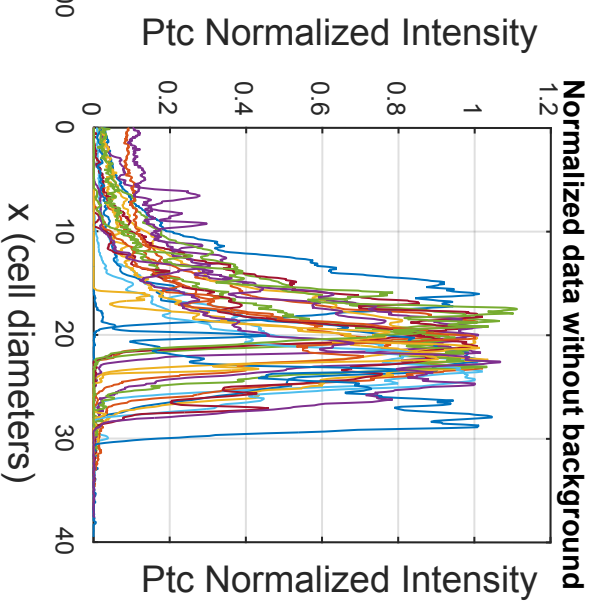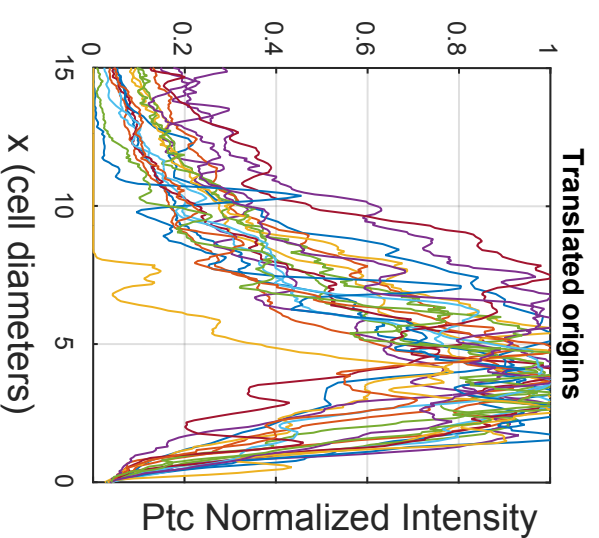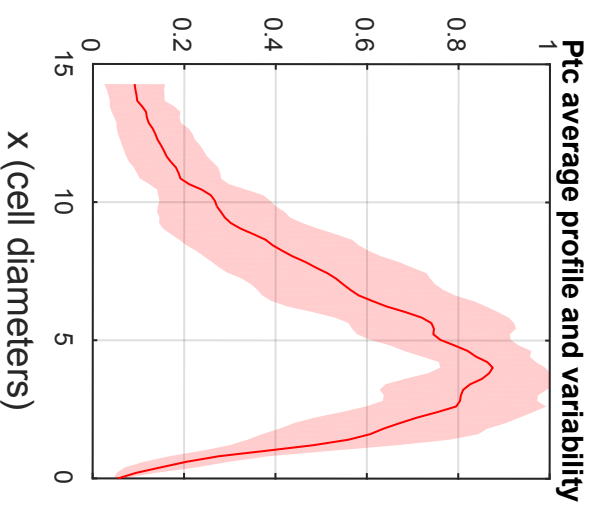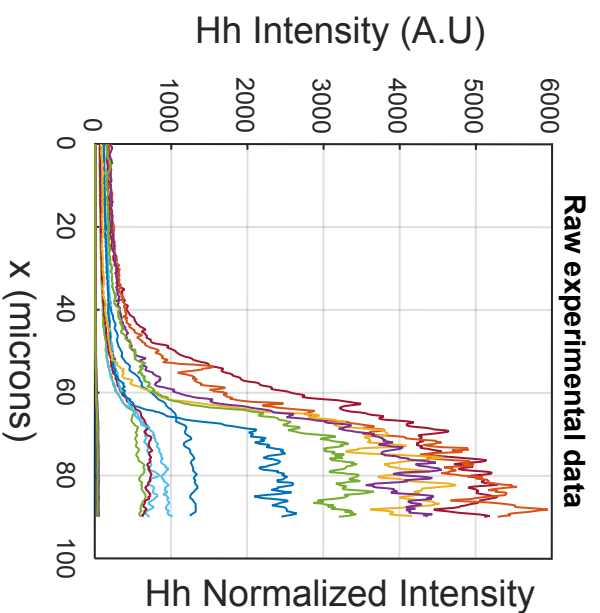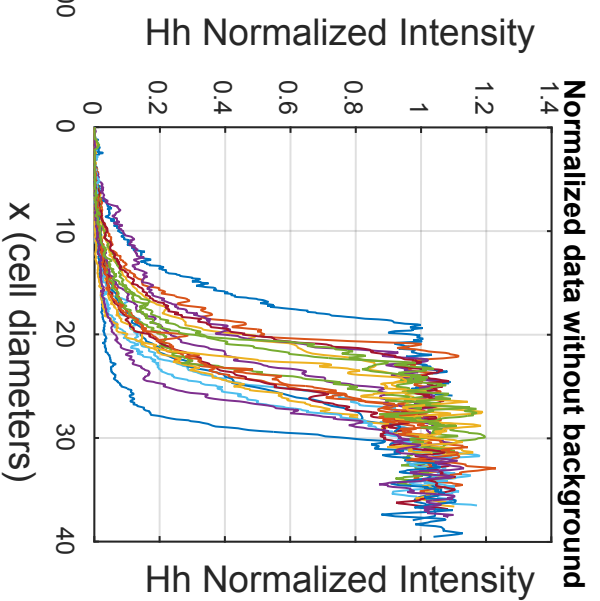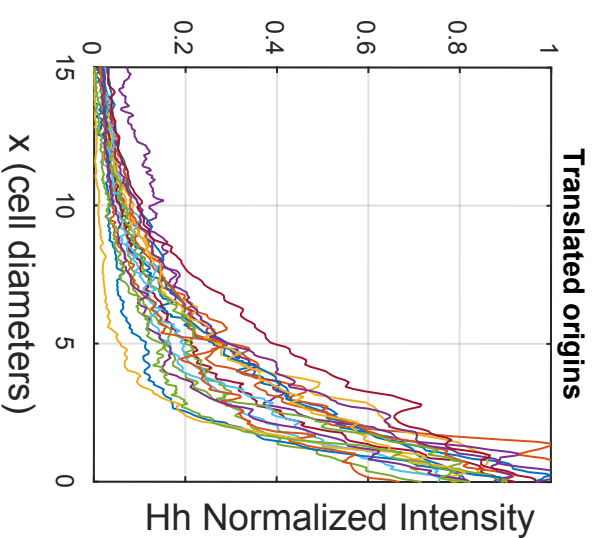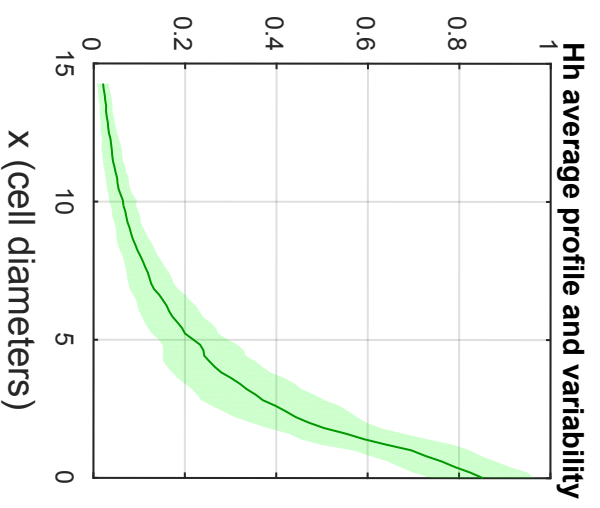

Supplement: S1 Fig — The normalization processes for Ptc (top) and Hh (bottom) profiles described in Materials and Methods are represented. The first column show the raw data obtained using the FIJI plot profile tool. The second column illustrates the normalized signal in cell diameters without background. The third column represents the same normalized data translated to the common origin at the A/P compartment border, using the beginning of Ptc expression to locate it. The final column shows the average (continuous thin lines) and the variability of the experimental samples (standard deviations are plotted in color shaded areas, Ptc in red and Hh in green). (PDF) [file pcbi.1009245.s006.pdf]

**A**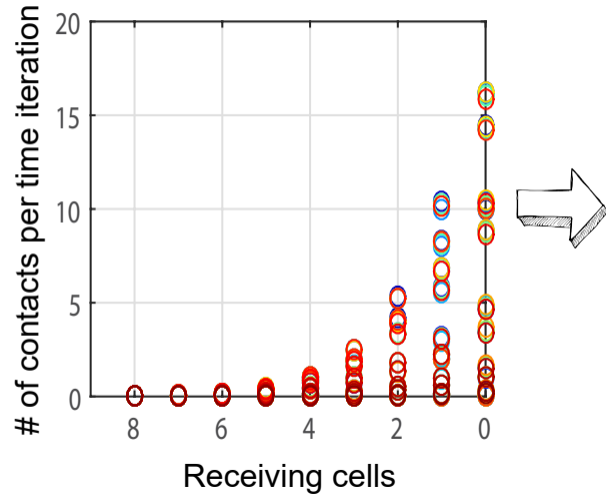**B**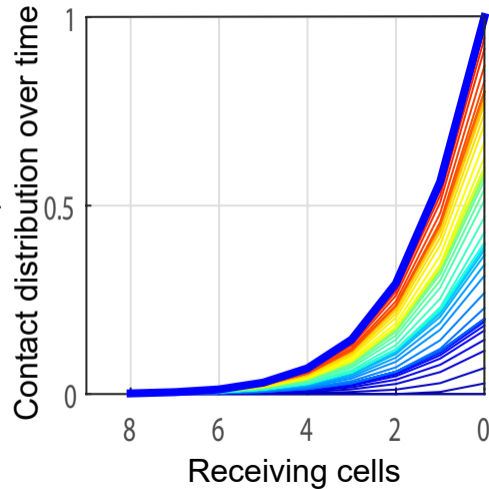**C**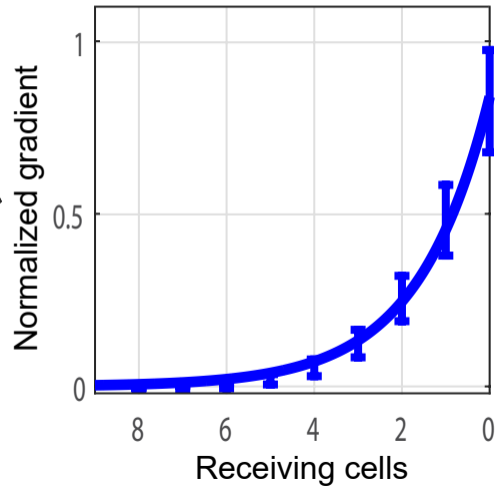

Supplement: S2 Fig — The grafts show that the spatial distribution of contacts in the receiving cells (A) together with its temporal evolution (B) determine the final shape of the gradient (C). (PDF) [file pcbi.1009245.s007.pdf]

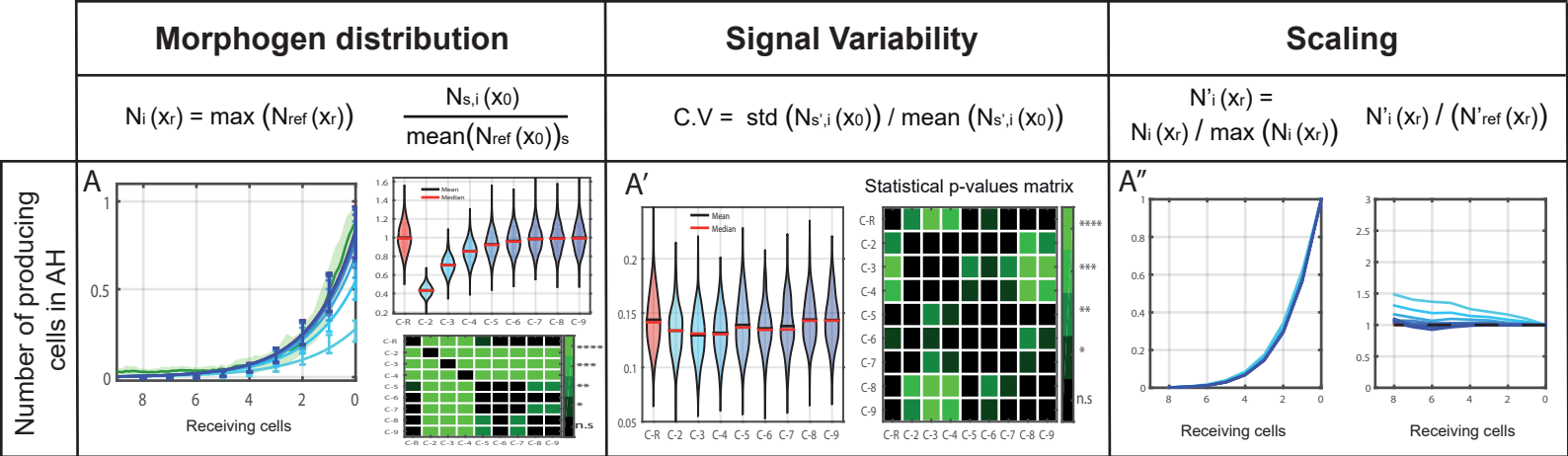

Supplement: S3 Fig — The grafts show how different number of producing cell rows change the gradient properties: amount of the transmitted morphogen (A), signal variability (A’) and scaling (A”). Since the length of the cytonemes and the size of the Hh producing cells in abdominal histoblast nets are smaller than those in imaginal wing discs, these results confirm the behavior observed in imaginal wing discs but with a scaling factor. (PDF) [file pcbi.1009245.s008.pdf]

**A**
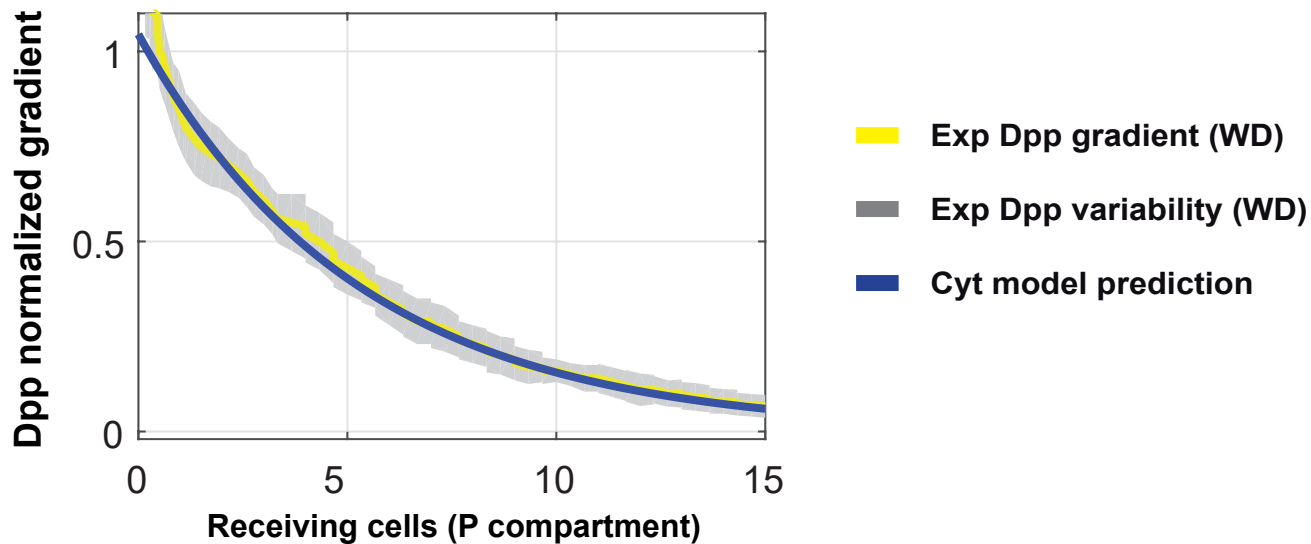
**B**
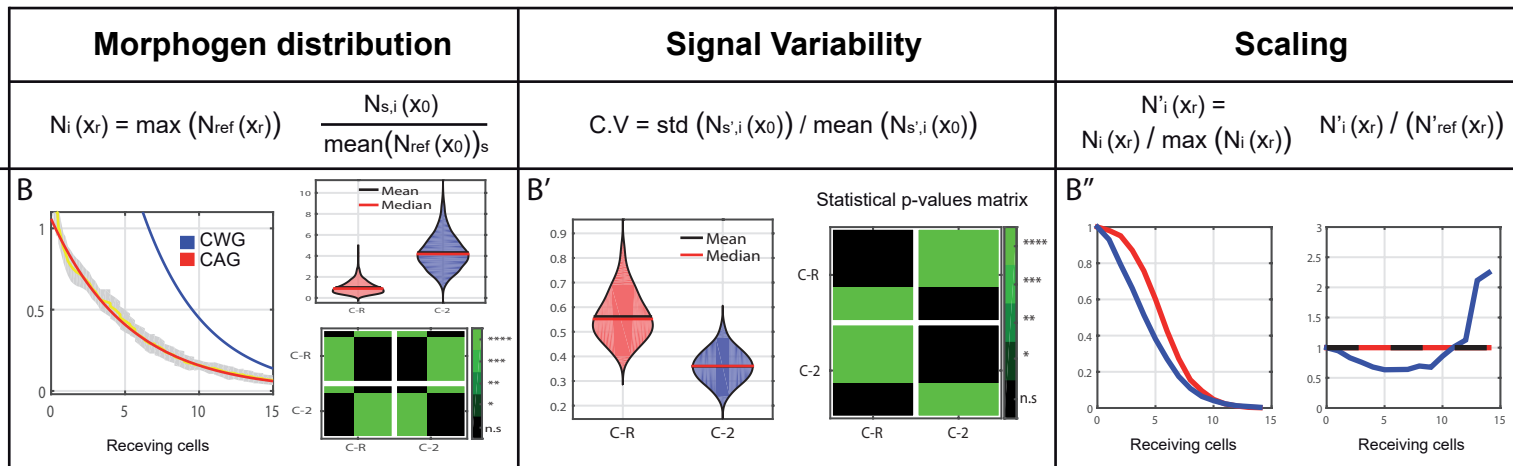

Supplement: S7 Fig — (A) Cytomorph simulations for Dpp gradient (blue) compared to the real experimental Dpp gradient [50] (average in yellow and experimental variability in grey). (B) Dpp cytonemes contacting while growing (CWG, blue) or contacting after growth (CAG, red). The simulations show that two types of dynamics predict relevant differences in the amount of morphogen transferred (B), the signal variability (B’) and the gradient shape (B”). Our results suggest that cytonemes contacting after growth fits better the experimental Dpp gradient. (PDF) [file pcbi.1009245.s012.pdf]
