# Supplementary material for: Improving the understanding of cytoneme-mediated morphogen gradients by in silico modeling
Source: PLoS Comput Biol. 2021 Aug 3;17(8):e1009245. doi: 10.1371/journal.pcbi.1009245 (PMC8362982; doi:10.1371/journal.pcbi.1009245)
Supplement: S4 Fig — These results show that increasing the number of A compartment cell rows does not change the gradient properties: amount of transmitted morphogen (A), signal variability (A’) and scaling (A”). These results are not surprising since the amount of Hh taken by each receiving cell of the A compartment only depends on the contacts of that cell with the producing cells of the P compartment. (PDF) [file pcbi.1009245.s009.pdf]

	Morphogen distribution	Signal Variability	Scaling
	$\frac{N_{s,i}(x_0)}{mean(N_{ref}(x_0))_s}$	$C.V = std\left(N_{s',i}(x_0)\right) / mean\left(N_{s',i}(x_0)\right)$	$N'_i(x_r) = N_i(x_r) / max\left(N_i(x_r)\right)$; $N'_i(x_r) / \left(N'_{ref}(x_r)\right)$
Number of anterior cells	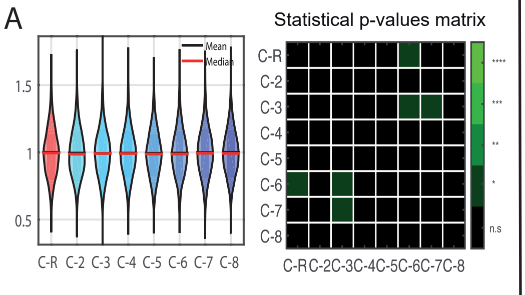	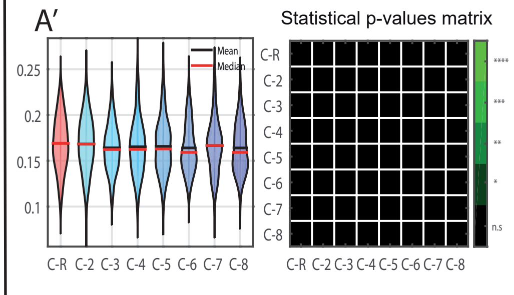	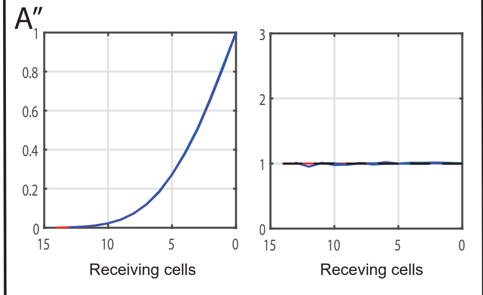
