# Supplementary material for: Improving the understanding of cytoneme-mediated morphogen gradients by in silico modeling
Source: PLoS Comput Biol. 2021 Aug 3;17(8):e1009245. doi: 10.1371/journal.pcbi.1009245 (PMC8362982; doi:10.1371/journal.pcbi.1009245)
Supplement: S5 Fig — (A) Simulations showed that dynamic and static cytonemes generate different gradient shapes. The differences suggest that cytoneme dynamics play a pivotal role in shaping the Hh gradient (A”). Simulations for static and dynamic cytonemes (dynamic with “tc = 60s” in red, static with “tc = 120s” in light blue, static with “tc = 300s” in dark blue) showed that the final amount of morphogen released depends on factors, such as the time taken for a contact to be effective (time-length contacts “tc”). (B) Growth simulations of triangular (t) and Trapezoidal (T) dynamics of cytonemes contacting while growing (= 1) or contacting at maximum elongation (= 0) (Ref.case T = t = 1 in red, case 2: T = 0,t = 1 in light blue, case 3: T = 1,t = 0 in blue, case 4: T = 0,t = 0 in dark blue). The simulations shown for contact during growth using each type of behavior, triangular or trapezoidal, result in similar effects without statistically significant differences (C2 and C3 in B, B ’and B’ ’). However, if both types of cytoneme behaviors happen together, either contacting while growing or just contacting after growth (C-R and C4 respectively), there are relevant differences in the amount of morphogen transferred (B), the signal variability (B’) and the gradient shape (B”). (PDF) [file pcbi.1009245.s010.pdf]

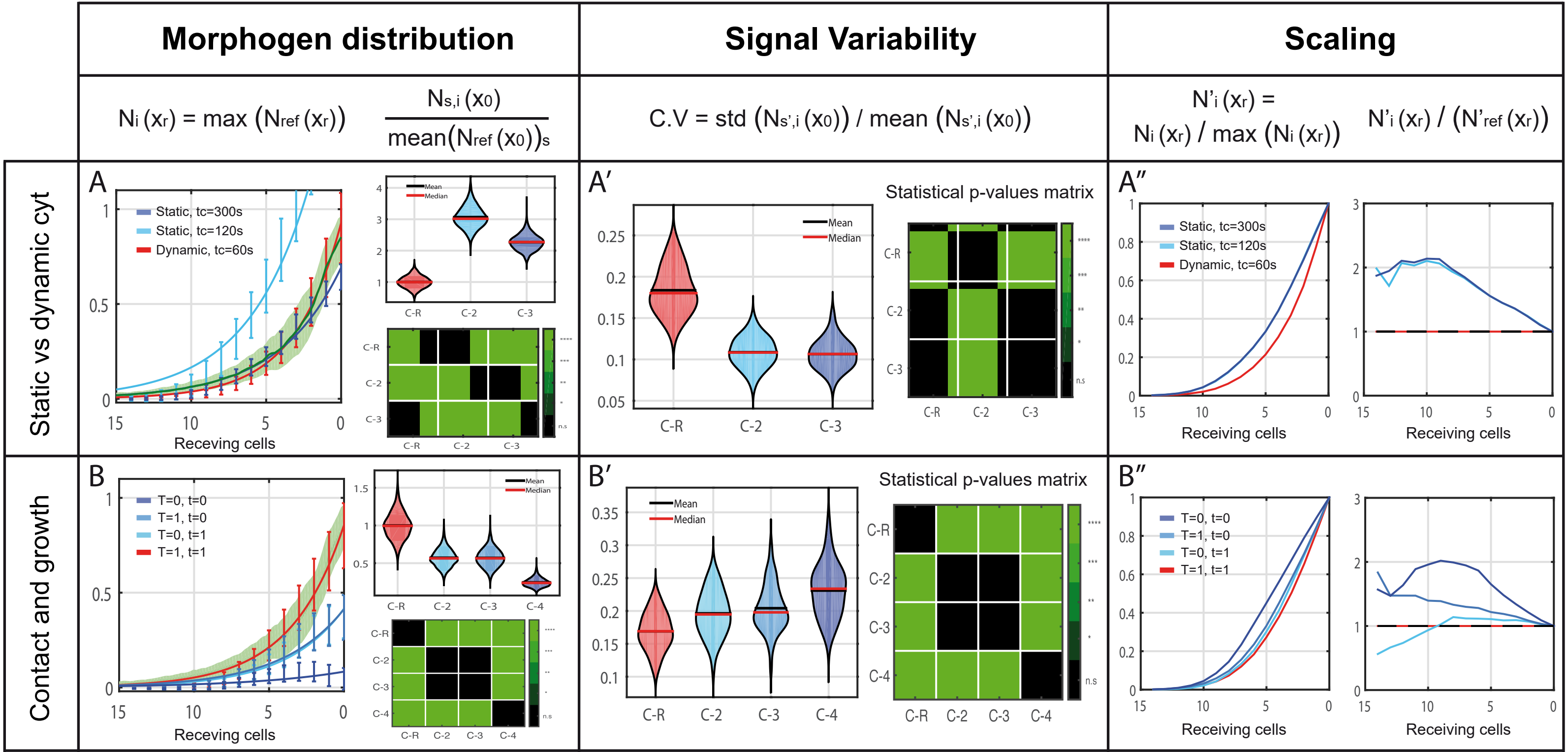
Morphogen distribution
Signal Variability
Scaling
Ni (xr) = max (Nref (xr))
Ns,i (x0) / mean(Nref (x0))s
C.V = std (Ns',i (x0)) / mean (Ns',i (x0))
N'i (xr) = Ni (xr) / max (Ni (xr))
N'i (xr) / (N'ref (xr))
Static vs dynamic cyt
Contact and growth
A
Static, tc=300s
Static, tc=120s
Dynamic, tc=60s
Receving cells
Mean
Median
C-R
C-2
C-3
A′
Statistical p-values matrix
A″
Receiving cells
B
T=0, t=0
T=1, t=0
T=0, t=1
T=1, t=1
Receving cells
C-4
B′
Statistical p-values matrix
B″
Receiving cells
