# Supplementary material for: Improving the understanding of cytoneme-mediated morphogen gradients by in silico modeling
Source: PLoS Comput Biol. 2021 Aug 3;17(8):e1009245. doi: 10.1371/journal.pcbi.1009245 (PMC8362982; doi:10.1371/journal.pcbi.1009245)
Supplement: S6 Fig — Experimental data are represented in green, reference simulation for a normal tissue (NP = 15 and ncyt = 4) in red, altered tissue with less producing cells (NP = 3 and ncyt = 4) in light blue and altered tissue with a compensatory mechanism for this cell number reduction (NP = 3 and ncyt = 7) in dark blue. (A) Left, morphogen distribution (y-axis) along the receiving cells normalized to the maximum value of the reference case. Right, violin plots for the number of contacts in the first row of receiving cell x0, normalized to the reference average value (2000 simulations per case). A’) Violin plots for the coefficient of variation (y-axis) per case (x-axis) in the first row of receiving cells (x0). A”) Left, distribution of contacts normalized to their maximum (y-axis) to compare the changes in the shape in the receiving cells. Right, coefficient of the previous normalized distributions of contacts (y-axis) to study the scaling in the receiving cells. (PDF) [file pcbi.1009245.s011.pdf]

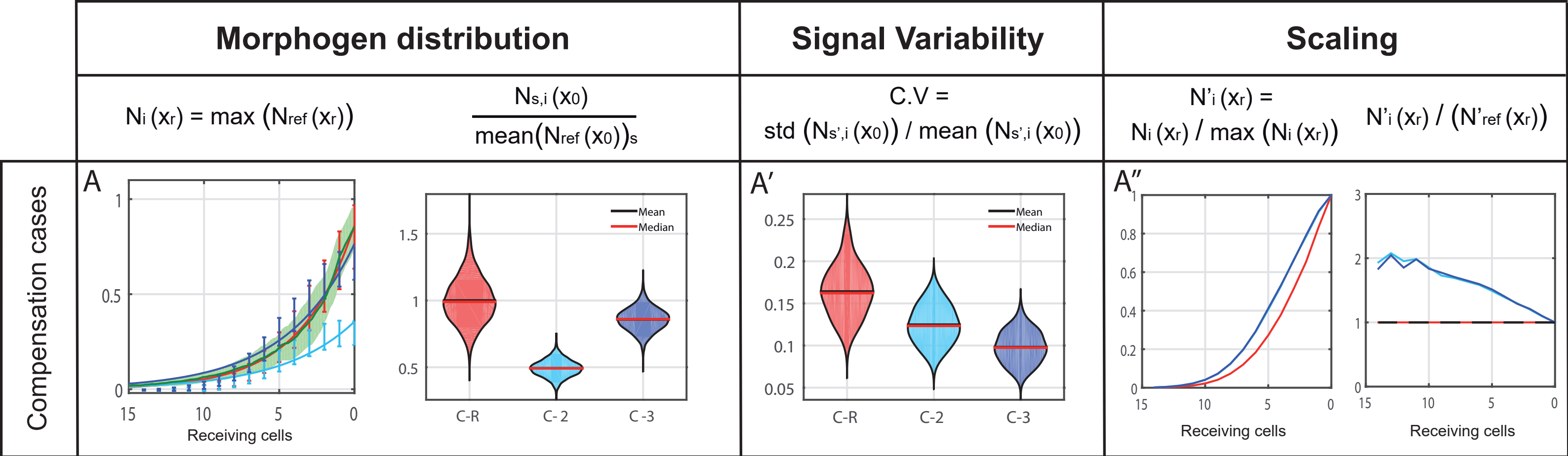

Morphogen distribution
Signal Variability
Scaling
Ni (xr) = max (Nref (xr))
Ns,i (x0) / mean(Nref (x0))s
C.V = std (Ns',i (x0)) / mean (Ns',i (x0))
N'i (xr) = Ni (xr) / max (Ni (xr))
N'i (xr) / (N'ref (xr))
Compensation cases
A
A'
A''
Receiving cells
Mean
Median
C-R
C-2
C-3
