# Supplementary material for: Improving the understanding of cytoneme-mediated morphogen gradients by in silico modeling
Source: PLoS Comput Biol. 2021 Aug 3;17(8):e1009245. doi: 10.1371/journal.pcbi.1009245 (PMC8362982; doi:10.1371/journal.pcbi.1009245)
Supplement: S8 Fig — Screenshot of the computational code used for the simulation of the diffusion-degradation model. Left) Main script with pdepe function for 1-D parabolic and elliptic PDEs. Right) Three auxiliary functions called by pdepe that contain: the diffusion-degradation equation (top), the boundary conditions (middle) and the initial conditions (bottom) as described in Material and Methods. (PDF) [file pcbi.1009245.s013.pdf]

Diffusion1D

```
function [u]=Diffusion1D
%% Simulation Parameters

P(1) = 0.033; % Diffusion coefficient D
P(2) = 1; %c0
P(3)=0.00007; % Degradation rate
P(4)=1; % Eponential decay of the degradation term

L = 45; %Length of domain
tmax = 3600; %Max. simulation time
phi=3; %Cell diameter conversion factor (micras/cell)

m = 0; %Parameter corresponding to the symmetry of the problem (see help)
t = linspace(0,tmax,100); %tspan
x = linspace(0,L,100); %xmesh


%% solving with PDEPE

sol = pdepe(m,@DiffusionPDEfun,@DiffusionICfun,@DiffusionBCfun,x,t,[],P);
u = sol;
x=x/phi;

figure;
hold all

for n = linspace(1,length(t),10)
plot(x,u(n,:),'LineWidth',2)

end

xlabel('Distance in cell diameters','fontsize',18,'fontweight','b','fontname','arial')
ylabel('Normalized concentration','fontsize',18,'fontweight','b','fontname','arial')
axis([0 L/phi 0 P(2)])
set(gca,'XDir','Reverse','FontSize',15,'fontweight','b','fontname','arial')
grid on
```

DiffusionPDEfun

```
function [c,f,s] = DiffusionPDEfun(x,t,u,dudx,P)
% Function defining the PDE
% Extract parameters
D = P(1);
deg=P(3);
n=P(4);
% PDE
c = 1;
f = D.*dudx;
s = -deg*(u.^n);
```

DiffusionBCfun

```
function [pl,ql,pr,qr] = DiffusionBCfun(xl,ul,xr,ur,t,P)
% Boundary conditions for x = 0 and x = L;
% Extract parameters
c0 = P(2);
% BCs: No flux boundary at the right boundary and
% constant concentration on % the left boundary
pl = ul-c0; ql = 0; pr = 0; qr = 1;
```

DiffusionICfun

```
function u0 = DiffusionICfun(x,P)
% Initial conditions for t = 0; can be a funciton of x
u0 = 0;
```
