# Supplementary material for: Improving the understanding of cytoneme-mediated morphogen gradients by in silico modeling
Source: PLoS Comput Biol. 2021 Aug 3;17(8):e1009245. doi: 10.1371/journal.pcbi.1009245 (PMC8362982; doi:10.1371/journal.pcbi.1009245)
Supplement: S1 Table — Numerical parameters used in the cytoneme model for each simulation. When a scan of a single variable is performed, the values are written from the initial to the final values with a specific step between them. Binary values mean that this condition is on = 1 or off = 0. WD and AH are abbreviations for wing imaginal discs and abdominal histoblast nests and T and t for Trapezoidal and Triangular behaviors respectively. Units are specified at the top of each column. (PDF) [file pcbi.1009245.s017.pdf]

S1 Table. Parameters used in cytoneme model simulations

Simulations and cases	N_R	N_P	Average Cell size Φ (μm)	Number of simulations	Probability of contact μ	Signaling type<br>Type1-2=0<br>Type3=1	Contact along overlap surface on?	Ncyt	Degradation rate (s^{-1})	Time of development to simulate (s)	Time of contact t_c(s)	Dynamic on?	ve triangle (cell/s)	vrt triangle (cell/s)	ve Trapezoid (cell/s)	vrt Trapezoid (cell/s)	Probability of having triangles p_t	Contact along growing on?	Exp data
Exp Vs Model WD	15	15	3	2000	[1;1]	1	1	4	$7 \cdot 10^{-5}$	3600	60	1	0.016	-0.014	0.019	-0.016	0.5	T=t=1	WD
Exp Vs Model AH	9	9	4.37	2000	[1;0.5]	1	0	4	$7 \cdot 10^{-5}$	3600	60	1	0.011	-0.010	0.013	-0.011	0.5	T=t=1	AH
Reference case	15	15	3	2000	[1;1]	1	1	4	$7 \cdot 10^{-5}$	3600	60	1	0.016	-0.014	0.019	-0.016	0.5	T=t=1	WD
Change in cell size	15	15	2.5 to 3.5 each 0.2	2000	[1;1]	1	1	4	$7 \cdot 10^{-5}$	3600	60	1	0.048/Φ	-0.042/Φ	0.057/Φ	-0.048/Φ	0.5	T=t=1	WD
Density of cytonemes	15	15	3	2000	[1;1]	1	1	1 to 3 each 1	$7 \cdot 10^{-5}$	3600	60	1	0.016	-0.014	0.019	-0.016	0.5	T=t=1	WD
Number of producing cells	15	1 to 14 each 1	3	2000	[1;1]	1	1	4	$7 \cdot 10^{-5}$	3600	60	1	0.016	-0.014	0.019	-0.016	0.5	T=t=1	WD
Number of receiving cells	2 to 14 each 2	15	3	2000	[1;1]	1	1	4	$7 \cdot 10^{-5}$	3600	60	1	0.016	-0.014	0.019	-0.016	0.5	T=t=1	WD
Compensation cases	15	3	3	2000	[1;1]	1	1	C2=4<br>C3=7	$7 \cdot 10^{-5}$	3600	60	1	0.016	-0.014	0.019	-0.016	0.5	T=t=1	WD
Type of signaling: Type 3	15	15	3	2000	[1;1]	1	1	4	$7 \cdot 10^{-5}$	1800	60	1	0.016	-0.014	0.019	-0.016	0.5	T=t=1	WD
Type of signaling: Type 1-2	15	15	3	2000	[1;1]	0	1	4	$7 \cdot 10^{-5}$	1800	60	1	0.016	-0.014	0.019	-0.016	0.5	T=t=1	WD
No overlapping cytonemes	15	15	3	2000	[1;1]	1	0	4	$7 \cdot 10^{-5}$	3600	60	1	0.016	-0.014	0.019	-0.016	0.5	T=t=1	WD
Probability function	15	15	3	2000	Linear decay	1	0	4	$7 \cdot 10^{-5}$	3600	60	1	0.016	-0.014	0.019	-0.016	0.5	T=t=1	WD
Probability of contact	15	15	3	2000	[0.4;0.4] to [0.8;0.8] each [0.2;0.2]	1	1	4	$7 \cdot 10^{-5}$	3600	60	1	0.016	-0.014	0.019	-0.016	0.5	T=t=1	WD
Overlapping cytonemes	15	15	3	2000	[1;1]	1	1	4	$7 \cdot 10^{-5}$	3600	60	1	0.016	-0.014	0.019	-0.016	0.5	T=t=1	WD
Contact and growth	15	15	3	2000	[1;1]	1	1	4	$7 \cdot 10^{-5}$	3600	60	1	0.016	-0.014	0.019	-0.016	0.5	C2:T=0,t=1<br>C3:T=1,t=0<br>C4:T=t=0	WD
FRAP simulation	7	7	4.37	2000	[1;0]	0	0	4	$7 \cdot 10^{-5}$	3360	60	1	0.011	-0.010	0.013	-0.011	0.5	T=t=1	AH
Cytoneme triang/trap dynamics	15	15	3	2000	[1;1]	1	1	4	$7 \cdot 10^{-5}$	3600	60	1	0.016	-0.014	0.019	-0.016	C2=0.1<br>C3=0.9	T=t=1	WD
Static vs dynamic: Dynamical case	15	15	3	2000	[1;1]	1	0	4	$7 \cdot 10^{-5}$	1800	60	1	0.016	-0.014	0.019	-0.016	0.5	T=t=1	WD
Static vs dynamic: static	15	15	3	2000	[1;1]	1	0	4	$7 \cdot 10^{-5}$	1800	C1=120<br>C2=300	0	0.016	-0.014	0.019	-0.016	0.5	T=t=1	WD
Number of producing cells AH	9	1 to 8 each 1	4.37	2000	[1;0.5]	1	0	4	$7 \cdot 10^{-5}$	3600	60	1	0.011	-0.010	0.013	-0.011	0.5	T=t=1	AH
Dpp exp Model WD	15	7	3	2000	[1;1]	1	1	4	$2.52 \cdot 10^{-4}$	600	60	1	0.019	-0.016	0.016	-0.014	0.5	T=t=0	WD
