# Supplementary material for: Improving the understanding of cytoneme-mediated morphogen gradients by in silico modeling
Source: PLoS Comput Biol. 2021 Aug 3;17(8):e1009245. doi: 10.1371/journal.pcbi.1009245 (PMC8362982; doi:10.1371/journal.pcbi.1009245)
Supplement: S2 Table — Statistical p-values used to compare elongation, retraction and stationary phases using a Kolmogorov-Smirnov statistical analysis (n.s = no significance). (PDF) [file pcbi.1009245.s018.pdf]

| k-s test | | Anterior | | | | |
|---|---|---|---|---|---|---|
| | | ts | te | | tr | |
| Posterior | ts | ns | Triangle | Trapezoid | Triangle | Trapezoid |
| | te | Triangle | ns | 0.027 | ns | 0.02 |
| | | Trapezoid | 0.015 | ns | 0.001 | ns |
| | tr | Triangle | ns | 0.0014 | ns | 0.011 |
| | | Trapezoid | ns | ns | 0.064 | ns |

| k-s test | | Anterior | | | | |
|---|---|---|---|---|---|---|
| | | ts | te | | tr | |
| Anterior | ts | | Triangle | Trapezoid | Triangle | Trapezoid |
| | te | Triangle | | 0.035 | ns | 0.044 |
| | | Trapezoid | | | 0.0028 | ns |
| | tr | Triangle | | | | 0.02 |
| | | Trapezoid | | | | |

| k-s test | | Posterior | | | | |
|---|---|---|---|---|---|---|
| | | ts | te | | tr | |
| Posterior | ts | | Triangle | Trapezoid | Triangle | Trapezoid |
| | te | Triangle | | 0.022 | ns | ns |
| | | Trapezoid | | | 0.0005 | ns |
| | tr | Triangle | | | | 0.04 |
| | | Trapezoid | | | | |
