# Supplementary material for: Improving the understanding of cytoneme-mediated morphogen gradients by in silico modeling
Source: PLoS Comput Biol. 2021 Aug 3;17(8):e1009245. doi: 10.1371/journal.pcbi.1009245 (PMC8362982; doi:10.1371/journal.pcbi.1009245)
Supplement: S3 Table — Numerical parameters used in the Fig 4 for diffusion simulations. Units are specified at the top of each column. (PDF) [file pcbi.1009245.s019.pdf]

Tissue	Diffusion constant: D(μm^2/ s^{-1})	Length of simulated domain (μm)	Cell diameter Φ (μm)	Developmental time (s)	Degradation rate: δ (s^{-1})	Average experimental maximum (Source normalization): $\langle u_{exp}^{N} \rangle_{x=0}$
**Wing disc**	0.033	48	3	3600	$7 \cdot 10^{-5}$	0.852
**Abdominal histoblasts**	0.033	39.33	4.37	3600	$7 \cdot 10^{-5}$	0.878
**Abdominal histoblasts**	0.011	39.33	4.37	3600	$7 \cdot 10^{-5}$	0.878
