# Supplementary material for: Improving the understanding of cytoneme-mediated morphogen gradients by in silico modeling
Source: PLoS Comput Biol. 2021 Aug 3;17(8):e1009245. doi: 10.1371/journal.pcbi.1009245 (PMC8362982; doi:10.1371/journal.pcbi.1009245)
Supplement: Attachment — Submitted filename: Response to Reviewers.pdf [file pcbi.1009245.s020.pdf]


Reviewer #1 (Evidence, reproducibility and clarity (Required)):

Summary:

In this work the authors present a simple mathematical model for the distribution of morphogen molecules that travel via cytonemes through a 1-dimensional system. This model is used as a basis for a software package called Cytomorph that takes as an input a set of experimentally measured distributions of cytoneme dynamics as well as experimenter determined parameters such as contact probability and method of cytoneme growth and retraction. The Cytomorph package then outputs spatial and temporal information on the distribution of morphogen as well as cytonemes and their contacts with cells and other cytonemes, all obtained over thousands of simulation runs. A number of in silico experiments are then performed to show that these outputs agree with experimentally measured morphogen distributions of Hedgehog in the imaginal wing disc and abdominal histoblast nest. Further in silico experimentation is done to study how this distribution is affected by a wide array of parameters such as producer row number, cytoneme connection method, and connection probability function. Comparisons to the traditional diffusion based model are also made. The authors find a suite of results based on these experiments and accordingly present the Cytomorph software package as a useful and adaptable tool for the community.

Major comments:

While the various in silico experiments present an expansive and exhaustive study of the different ways in which Cytomorph can be used to examine a cytoneme based distribution system, the machinery behind the software is left notably underdescribed. The authors do not sufficiently make clear what exactly happens within each iteration of the simulations run by Cytomorph, leaving the results irreproducible without the reader going into and deciphering the software code itself.

In order to improve the description of the mathematical and computational steps behind the software, we have created a visual organigram (new Supplementary Figure S.1) with a detailed depiction of the steps. We have also

included a short description in the main text and an extended explanation in the Supplementary Material section.

Some of the specific details left undiscussed are how it is determined when and where a cytoneme will spawn or what its maximum length will be, the dynamics of morphogen transport within the cytonemes, the effects of one cytoneme making multiple connections on how much morphogen is delivered through each connection, and where exactly stochasticity is introduced so as to allow for variations between simulation runs; amongst others.

In the new description of the software steps, we have tried to address the Referee's comments about the dynamics and stochasticity in more detail. In order to help the understanding of the variables, we have also tried to improve their description in the main text.

Additionally, when the authors investigate the diffusion model their stated boundary conditions do not match those presented at the end of the Materials and Methods section. The initial condition u(x,0)=0 and boundary condition du(L,t)/dt=0 represent a perfectly absorbing molecule sink at the x=L end of the system, not the reflecting boundary condition du(L,t)/dx=0 that would correspond to a zero morphogen flux.

We thank the Referee for noticing this annotation mistake since the equation is really dx instead of dt. We have corrected this error and included in the Supplementary Materials the exact lines of code used in Matlab pdepe to certify the conditions used in the resolution of the diffusion equation (new Supplementary Figure S.10).

Finally, while the authors spend a great deal of effort analyzing signal variability between simulation runs, there is no effort made to account for the inherently stochastic nature of molecular production, movement, and degradation. Particularly if molecule numbers are small, fluctuations in these processes could greatly increase signal variability. The authors should either address why these fluctuations are negligible or include them in the modelling.

This work is mainly focused on the transport of the morphogen; other terms as degradation were introduced directly using published experimental data. Regarding the main concern about the negligibility of the fluctuations for cytoneme transport, we agree with the Referee on the importance of this point. Therefore, we have included a detailed description of the variability and

fluctuations in a new section of the Supplementary Material. To help its understanding, we have also included a new Supplementary Figure (Supplementary Figure S.11).

The largest fluctuations were found at the tail of the morphogen gradient (last rows of receiving cells). Since this corresponds to the region where the amount of morphogen is low, the absolute fluctuations do not change the activation of the low-threshold target. We then conclude that those fluctuations are biologically negligible for our study.

Minor comments:

The authors should double check all equation and figure references as I noted several instances in which it appeared that the wrong equation or figure was being referred back to. Similarly, the authors should double check the equations themselves, particularly those in the supplemental material.

We thank the Referee for noticing these mistakes. We have reviewed those references in order to fix the wrongly linked ones.

Eqs. SM1.1 and SM1.2 have a plethora of parameters with a wide array of different sub- and superscripts that are left unexplained and possibly incorrectly labelled in some cases,

Equations SM1.1 and SM1.2 described a general form of Triangular and Trapezoidal dynamics and the different sub- and superscripts come from the published experimental data. Nevertheless, in order to make them more intuitive we have simplified the expressions and included a more detailed description of those parameters and their scripts in the revised version.

*while the second line of Eq. SM2.2 is nonsensical unless r_I*p=0 and p_i<=1.*

We thank the Referee for noticing the uncertainty in this equation, since it was written in an iterative syntax as it is coded in the software. Therefore, in the code we did not have this nonsensical range of data, but we agree that it should be specified with a mathematical syntax as the rest of the equations in the manuscript. Therefore, we redefined the notation and specified better the numerical domains of those variables.

Additionally, the notation used in Figs. 5 and 6 as well as the bottom part of Fig. 7 is confusing. The caption should more explicitly state what the various expressions in the second row of each column represent.

The second row represents the statistical analysis between cases coded in a color matrix, as it is described in the footnote. We thank the Referee for this recommendation because this is not the usual representation. Therefore, we have changed the previous explanation to one hopefully clearer and intuitive; we have also included a specific label in the figures.

In Fig. 5A specifically it is unclear what exactly the variable phi represents.

Phi is a widely used annotation in biology to define cell size diameter and cell position. We didn´t realize it could be unclear. For a better understanding within a multidisciplinary field we have changed this symbol.

Does it have anything to do with the phi that is used as a position variable for the cells, and if it is a ratio of cytoneme length to cell diameter then why does it have units of microns?

We agree that this phi notation is confusing. It has been used to indicate distance position as well as cell diameter. Although these variables are biologically related, in the new version of the manuscript we have changed the notation to separate both concepts and avoid misunderstandings.

Reviewer #1 (Significance (Required)):

As the Cytomorph model and software can be applied to a wide variety of systems involving morphogen transport via cytonemes, it provides a technical advance in our ability to analyze and discuss the results of measurements on cytonemes in a more homogenous way. This work and the resulting software is particularly applicable to and build off of studies done by other groups that study the dynamics of cytonemes such as the Kornberg lab (works from which are cited by the authors) and the Scholpp lab (such as Stanganello E, Scholpp S. Role of cytonemes in Wnt transport. J Cell Sci. 2016; 129(4):665-672), and as such it is experimental labs such as these that will be the most interested in this manuscript and its findings.

My field of expertise lies primarily in stochastic modeling and linear response

theory. As such, I feel I do not have sufficient expertise to evaluate the experimental methods outlined in this manuscript and determine their level of scientific rigor.

Reviewer #2 (Evidence, reproducibility and clarity (Required)):

The manuscript "Improving the understanding of cytoneme-mediated morphogen gradients by in silico modelling" addresses the role of in silico modelling in understanding pattern formation via cytonemes: filopodia that transport signalling molecules to and from cells. Investigating the role of cytonemes and, in particular, their dynamics, during development is an important and emerging field in developmental biology, and there is great potential for mathematical modelling to aid in understanding these processes.

The present manuscript attempts to derive a general set of equations describing pattern formation in the context of cytonemes, akin to that of the classic Turing model of morphogenesis. The authors replace the standard diffusion term in the PDE with a non-local term, intended to represent transport via cytonemes. This model is then posed over a one-dimensional domain with a source at one end and no flux boundary conditions at the other and is shown to be able to generate a morphogen gradient profile that could pre-pattern a biological tissue. The model is tested against a key experimental system, namely, Hh signalling in the Drosophila wing imaginal disc and is shown to reproduce some experimental results. Finally, the authors have developed a Matlab-based software package that they claim will be applicable to a wide range of systems. This GUI-based software allows users to input experimentally measured averages of cytoneme properties and explore the effect of these properties on tissue patterning.

My primary concern is that the paper presents itself as a mathematical model of cytoneme formation in general. The authors themselves state in their introduction that the mechanisms for cytoneme generation and maintenance are presently unknown. In fact, it is not even known if they are consistent across biological systems (and in fact, are probably not in general). As such, any present instantiation that connects cytoneme dynamics to tissue patterning can only hope to be specific to a particular system (in this case, the Drosophila wing imaginal disc.

As mentioned in the introduction, the connection of cytonemes with patterning has been described in several works. We had included a list of publications describing the implication of cytoneme-mediated signaling for several morphogens (FGF, Egf, Hh, Dpp, Wnt or Notch) and in many vertebrate and invertebrate systems (Drosophila, chicken, Xenopus, Zebra fish, mouse and human tissue culture cells).

Whilst one may use general models (like the heat equation) to study pattern formation since it requires only specification of parameters, the model here requires specification of families of functions, that are likely to differ from context to context and so the model is not general.

Our model inputs are parameters determined experimentally rather than families of functions. This misunderstanding might derive from the use of triangular and trapezoidal dynamics, which are equations included in the software code but not input functions. To avoid this confusion, we have specified the input data in tables S.1 and S.2 and clarified in the main text that the triangular or trapezoidal family of functions are just the names for the basic dynamics of cytonemes (triangular for elongation and retraction, and trapezoidal when there is a stationary phase in between).

Ultimately, the model is a statistical modelling framework masquerading as a mechanistic one.

In this work, we have not specified the mathematical area to which the model belongs. Furthermore, we always explicitly described the different variables and functions modeled. Therefore, we do not understand what the supposed masquerade is.

As further evidence of the lack of generality of the model, the studied domain is only one dimensional and has signalling sources at one end. This scenario is perfectly adequate for theoretical explorations of pattern-forming systems but is highly unlikely to capture the geometrical intricacies of real-world systems (and I note that even in the diffusive case, boundary conditions are critical for understanding what patterns ultimately arise for a given system).

We agree with the Referee that there are cases in biological systems in which it is required to work in 2D or even 3D to have a full comprehension of the process. Nevertheless, those are mainly related to biological patterns rather than to biological signaling gradients, which usually are studied (experimental and theoretically) in 1D. Therefore, we have limited our model to this case and

compared our in silico results with the published experimental data. In any case, we have emphasized in the text that our model is limited to signaling gradients with the source at one end, which is the case of the best studied morphogens: Hh (Sonic-hh), Dpp (BMP) or Wg (Wnt).

Actually, as prove of the generality of the model, we have predicted different properties of Dpp and Wg gradients using our model. We then validated the simulated results using the experimental data obtained from independent publications.

To simulate their model, the authors need to specify triangular and trapezoidal functions, which are unlikely to be generalisable to all contexts. As such, the model is not general and, in particular, there is no way to change the software to make it so.

Cytonemes are filopodial structures based on actin filaments that polymerize and depolymerize to elongate and retract. This is a general process for all filopodial structures and it is why cytonemes were classified in a previous published work as a triangular behavior or, if this dynamic has a stationary phase, as a trapezoidal behavior (Gonzalez-Méndez et al., 2017). Therefore, these functions are just a categorization introduced to better describe the intrinsic dynamics of cytonemes, that could be applied to most of the experimental cases. To attend this Referee's concern, we have included in the introduction a more detailed description of these behaviors, as well as the references of publications describing the dynamic behaviors of cytonemes for different morphogens and in different organisms.

Trying to make a generalization for all cases, we included in the model those situations in which the cytonemes were static rather than dynamic (detailed simulations comparing dynamic and static cases can be found in the old Supplementary Figure S.5 A (now S.7 A)).

We have concluded that the model can be considered generalizable since it includes the simplest and most general cases in terms of cytoneme dynamics.

Whilst the development of a GUI for this scenario is a nice contribution, I feel that the lack of generalisability will, at best, mean that the software enjoys little use, and at worst, may lead researchers unfamiliar with the modelling context to misuse it in error.

Once we knew the model could be generalized, we were concerned about the misuse of the mathematical model, and that was the reason why we decided to develop a GUI as simple as possible.

Furthermore, in the online repository there is, together with the open software, an user guide of Cytomorph with a full description of parameters, variables and outputs and how to use them properly.

In my opinion, this work would be better suited as a presentation of specific mathematical modelling of tissue patterning in the Drosophila wing imaginal disc. In this case, many of the above concerns would be addressed.

We have rewritten part of the text to indicate the limits of the model and make clear that it has been tested experimentally for the Hh pathway and in two different developing systems: wing imaginal discs and abdominal histoblast nests.

As evidence of a more general use of Cytomorph, we have added in the revised version of the manuscript a new section focused on data prediction for the gradients of Dpp and Wg. We have also included supplementary figures that validate the predictions of our model using published experimental data.

That said, there are still a number of issues with the presentation of the model and results. I shall detail these in the bullet point list below:

1. The domain for Eq. 1 needs to be made explicit. Later, it appears that the domain is a closed one-dimensional interval, but the use of arrows here implies that x is a vector and hence $x \in D \subset Rn$ with $n > 1$.

We initially described the general equation for morphogens as $x \in \mathbb{R}^n$ and later we limited it to 1D. This is why at the beginning x, as a vector, contained an arrow, although later it was a scalar variable. Since we were interested in 1D in this work, to avoid this kind of misunderstanding we have rewritten from the beginning the equations as 1D and clearly specified the x domain used: the set of natural numbers $x \in \mathbb{N}_0$.

2. It is unclear over what the sum in Eq. 2 is being taken.

The sum in Eq. 2 is over the number of producing cell rows. We have changed the notation to clarify this point.

3. The statement "we used the discrete cell position $x = \varphi$ as spatial coordinate" is vague and does not help the reader understand the discretization.

The number of cell diameters is a widely used discrete unit for position in Developmental Biology. As we expect the readers of this publication to be multidisciplinary, we have changed the notation to avoid misunderstandings and clarify this discretization.

4. p is used both as a probability and as an index for producer cells. This is confusing.

We have changed the notation to avoid misunderstandings.

5. As previously stated, the choice of trapezoidal/triangular cytoneme dynamics is not general. More work needs to be done to showcase how the authors came to the conclusion that this is the best choice, and how the functions (and their associated parameters) describing them were selected.

The names triangular and trapezoidal stand for the published dynamics for elongation and the retraction of cytonemes and we already argued about its generality. As we specified in the manuscript, these types of behaviors have been experimentally observed and, therefore, we considered that the experimental observation was reason enough to include them in the model. If more details are required, the Material and Method section and the Supplementary Table S.3 show that the times measured for triangular and trapezoidal dynamics are statistically different and, consequently, both behaviors have to be considered.

As mentioned in the manuscript, the associated parameters represent the times and velocities for the elongation or retraction that have already been thoroughly analyzed and published (González-Méndez et al., 2017). The question of the Referee about how these functions affect the gradient is answered in the text and in Figure 7 F.

6. I can see how Type 1 and Type 2 cytonemes could be expanded naturally to a higher dimensional case, but it is not clear how Type 3 cytonemes could be, since the probability of any two cytonemes occupying the same space in higher dimensions is likely to be small (if they are imbued with independent dynamics).

We agree with the Referee on this point. It is something that shall be considered for future improvements of the model in higher dimensions. For instance, a complex scenario in 2D will be required of a cytoneme guiding

model. Nevertheless, since the present study is limited to 1D, this concern is not applicable for the current model.

7. The statement: "the distance between cells must be smaller than, or equal to, the maximum length of the cytonemes" seems inconsistent with the equations below since λ(t) does not appear to be a maximum length.

The length of the cytonemes is controlled as a dynamic function described by λ(t). Our statement referred to the maximum length for each time step that is given by λ(t). We agree that the initial statement could lead to misunderstanding, so we have suppressed the word "maximum".

8. I think the authors are confusing probabilities and rates in their discussion of the model. Eq. 1 is a density model and so calling events probabilities here is slightly misleading. As a more general statement, I am currently interpreting contact function C as one defined as a rate, rather than as a set of probabilistic terms. If the latter is true, then Eq. 1 is invalid since it mixes processes at different levels of description.

We thank the Referee for this comment. We have studied in depth this observation but we could not exactly find why the Referee considers that the model is working at different levels. Even though we could not find where in the text we called "probabilities" to the events of eq1, we rewrote the text to make clear what we consider either probability or rate. In addition, in the Supplementary Material section we clarify how the model works and at what levels of modeling we are working.

Reviewer #2 (Significance (Required)):

In general, the paper is well written, however, the focus of the findings should be on patterning within an epithelium such as the Drosophila wing imaginal disk.

The work will be interesting for the developmental biology community as well as for the upcoming biomathematical modelling community.

Expertise: Developmental biologist with experience in tissue patterning and morphogen gradients

Referees cross-commenting

I agree with Reviewer 3 that the importance of cytoneme-mediated signalling has been described in several systems - invertebrates and vertebrates. However, I think the focus of this work in particular should be on cytoneme signalling in the wing imaginal disc. IMO, this would not limit the conclusion but rather focus it and make it then applicable to epithelial tissues in general. I agree with the other point.

Reviewer #3 (Evidence, reproducibility and clarity (Required)):

There is much to like in this thoughtful and worthwhile study that develops mathematics to describe how cytonemes might generate experimentally observed Hh gradients. Two suggestions:

1) I am not equipped to evaluate the mathematics and as a non-expert would find it helpful if the authors explicitly stated at the outset what assumptions they took, the specific contexts they sought to model, and the parameters that they explored.

We agree with the Referee on the excessively mathematical focus of our interpretation of the results in the old version of the manuscript. We have rewritten part of the text to clarify the biological implications of the variables and simulations explored.

Am I correct that they assume that the Hh gradient correlates with a cytoneme gradient, that all cytoneme contacts have the same duration and exchange equivalent amounts of Hh, and that the variables that were characterized are cytoneme length distributions, cytoneme extension rate, contact duration, and cytoneme density?

Since the mechanism of morphogen exchange is not fully identified, we assumed the simplest case in which all the contacts have the same duration and exchange the same amount of morphogen. Using this approach, we were able to reproduce the gradient and concluded that it is not strictly necessary

to propose a more complex mechanism to establish a graded distribution of morphogens. We therefore worked under this assumption.

The variables characterized were the ones pointed out by the Referee, mainly cytoneme features, as the cytoneme length distributions or the different parameters of the temporal dynamics. We tried to define better these variables in the new version of the manuscript.

2) One of the unusual features of the Hh gradient in the wing disc is that the size of the posterior compartment field of Hh-producing cells is large relative to the size and extent of the Hh gradient in the adjacent anterior compartment. Wing discs with large hh mutant clones, wing discs with large smo mutant clones, and wing discs with ttv mutant clones that block Hh uptake provide evidence that the Hh gradient is constituted with Hh that is produced by many cells, some that are far from the compartment border as well as some that are close. Has this been factored into the author's model?

Indeed. Being aware of the importance of the size of the signal source, we simulated how changing the size of the posterior compartment affects the gradient (altering the number of producing cell rows involved, figure 5B). In the old version of the manuscript we had focused on the theoretical approach, so we thank the reviewer for noticing that we should introduce a more biological point of view. Therefore, we included in the revised version of the manuscript a biological interpretation of how our simulations can help to understand the question posed by the reviewer.

Does the fact that the relative size of the posterior compartments and Hh gradients in the histoblasts is not as extreme as it is in the wing disc influence their model?

Following the Referee's question, we decided to simulate the influence of the relative size of the posterior compartment in the abdominal histoblast nests. We found that in both wing discs and histoblasts, the size of the posterior compartment affects the gradient but in a different scale factor. We have included these data in the revised version of the manuscript (new supplementary figure S.5).

Interestingly, this feature of the Hh gradient in the wing disc is not shared with other gradients in the wing disc such as the Wg, Dpp, and Bnl gradients. I would be interested to know if the author's model can be queried to suggest what properties might contribute to this difference?

In order to answer the reviewer question, we have used our model to tentatively simulate Wg and Dpp gradients. Our preliminary results suggest that considering only cell position and cytoneme length, the Wg and Dpp gradient lengths can be predicted in wing imaginal disc. Nevertheless, each morphogen has its own particularities and further studies are required for a precise simulation of these gradients. We included these results in a new section of the manuscript and in the new Supplementary Figure S.9.

Reviewer #3 (Significance (Required)):

This is an important contribution to gaining a basic understanding of the role of various properties of dynamic cytonemes to gradient formation.

Referees cross-commenting

I discount the apparently strongly held opinion of Reviewer #2 that "it is not even known if they [cytonemes] are consistent across biological systems (and in fact, are probably not in general)". I do not know where this comes from and do not think that such opinions are appropriate for anonymous reviews.

Cytoneme-mediated signaling has in fact been observed and characterized in many diverse biological systems. I submit that in contrast, mechanisms of dispersion based on diffusion are inferred and lack direct experimental evidence. I do agree that it is fair to ask the authors to carefully describe their work in the context of epithelial signaling, but it is not correct to ask them to limit their conclusions to the wing disc as the authors analyze both wing disc and histoblast signaling. They clearly state that their work is limited to 1D and so we understand that it is inadequate to model 3D morphologies. I do not criticize them for this.
