# Supplementary material for: Improving the understanding of cytoneme-mediated morphogen gradients by in silico modeling
Source: PLoS Comput Biol. 2021 Aug 3;17(8):e1009245. doi: 10.1371/journal.pcbi.1009245 (PMC8362982; doi:10.1371/journal.pcbi.1009245)
Supplement: Attachment — Submitted filename: Response to revierwers.docx [file pcbi.1009245.s021.docx]

Reviewer #1: In this version of the manuscript the authors have sufficiently addressed most of the concerns I raised during the first round of reviews. However, there are still important details about the physical mechanisms being modeled that remain absent. Most importantly, the dynamics of how either producing or receiving cells create new cytonemes is left critically underdescribed.

How new cytonemes are generated is still an open question that has not yet been solved experimentally. Some works have described the actine nucleation sites and how actin is organized to create new filopodial structures. The description of these processes focused on the molecular complex that initiates the nucleation and the birth of a filopodium, which is a different level of description from that of our model.

The GUI interface has a parameter labelled "Number of cytonemes per cell", which I presume is the number of cytonemes each cell is intended to grow, but this does not appear to be strictly stated.

Indeed, the parameter “number of cytoneme per cell” considers independent cytonemes protruding from the same cell. We have tried to make clear the description of this parameter.

Additionally, after a cytoneme completes its life cycle of growing and retracting and possibly pausing in between is it immediately replaced by a new cytoneme that undergoes the same dynamics? If not, is there some form of time lag between one cytoneme fully retracting and another beginning its extension from the same cell and what determines this time lag?

After a life cycle of a cytoneme another cytoneme can be created independently of the previous one, therefore it could initiate a different cycle of growing and retracting. Regarding to the renewal time-lag, there are not experimental data, for that reason we assumed a temporal homogeneous distribution of cytonemes along the simulation that is controlled by the parameter “Temporal density”, which determines the frequency of creation of cytonemes per cell in the model. We have rewritten this point in order to make the dynamics of temporal renewal understandable.

Given that an explicit understanding of the dynamics of the birth and death of cytonemes is crucial for the existence of a well-defined steady state morphogen distribution profile, it should be clearly outlined in the manuscript.

Since the explicit understanding of the dynamics of the birth and death of cytonemes is not well known experimentally, we have made a theoretical approximation summarizing this dynamic in a single parameter named “temporal density” that determines the frequency of cytoneme creation.

A further question that is immediately raised once the birth-death dynamics of the cytonemes within the model is established is how do these dynamics compare to those observed in the experiments. What are the statistics of how many cytonemes each cell has active at any given time or has such a distribution not been sufficiently measured?

As we have mentioned there is no direct measure of this cytoneme feature and, therefore, it is currently not possible to make a comparison.

A discussion as to how frequently cytonemes are created and removed from the system and its effect on the number of cytonemes each cell currently has extended would not only provide a useful understanding of how accurately the model used in Cytomorph is replicating this distribution but could also potentially introduce a path towards more precise measurements being made in the future to better understand and more precisely simulate these dynamics.

We also agree on the importance of this property to understand the dynamics of morphogen gradients. However, the lack of experimental data makes this study difficult and a pure theoretical approach would require further work. Consequently, we believe that this issue should be addressed in future works.

Finally, the authors should make sure to carefully spell check the manuscript. I noticed several places in the Discussion section where pronouns appear to have been dropped and the word "established" was misspelled as "stablished".

We have reviewed the manuscript and corrected the spelling mistakes.

----------------------------------------------------------------------------------------------

Reviewer #2: I commend the authors for their work and have only one request: the data showing that cytonemes move morphogens between cells and are responsible for morphogen exchange between producing and receiving cells is very strong. This is not therefore "a model" and should not be described as a "proposal".

The terminology “model” is normally used in a theoretical and mathematical approximation for in silico modelling of a biological process. We want to thank reviewer 2 to notice this possible misunderstanding and we tried to emphasize this and all the experimental evidence supporting cytoneme-mediated signaling.

----------------------------------------------------------------------------------------------

Reviewer #3: The manuscript has strongly improved, all my suggestions and comments have been addressed. Therefore, I support publication of the manuscript.
